# Psychometric properties and utility of the Responses to Positive Affect questionnaire (RPA) in a sample of people with bipolar disorder

**DOI:** 10.1002/jclp.22819

**Published:** 2019-06-25

**Authors:** Jannis T. Kraiss, Peter M. ten Klooster, Melissa Chrispijn, Anja W.M.M. Stevens, Ralph W. Kupka, Ernst T. Bohlmeijer

**Affiliations:** ^1^ Department of Psychology, Health, and Technology Center for eHealth and Well‐being Research University of Twente Enschede The Netherlands; ^2^ Center for Bipolar Disorders Dimence Mental Health Deventer The Netherlands; ^3^ Amsterdam UMC Department of Psychiatry Amsterdam Public Health Research Institute Vrije Universiteit Amsterdam The Netherlands

**Keywords:** bipolar disorder, dampening, emotion regulation, positive rumination, well‐being

## Abstract

**Objectives:**

To evaluate the psychometric properties of the Responses to Positive Affect (RPA) questionnaire in a sample of persons with bipolar disorder (BD).

**Method:**

Cross‐sectional survey study with 107 persons with BD. The original 3‐factor model of the RPA was compared with a 2‐factor model. Construct validity was determined with measures of well‐being, personal recovery, social role participation, and psychopathology and incremental validity was evaluated.

**Results:**

The fit of the 3‐factor model was acceptable for most fit indices. Subscores of the RPA revealed a significant relationship with aspects of well‐being, personal recovery, and psychopathology. Dampening and self‐focused positive rumination explained additional variance in personal recovery above and beyond well‐being.

**Conclusions:**

The RPA is an internally consistent and valid tool to assess positive emotion regulation processes in persons with BD. Specifically, the processes of dampening and emotion‐focused positive rumination seem to play an important role in BD.

## 1 INTRODUCTION

Bipolar disorder (BD) is a chronic mood disorder characterized by recurring depressive and (hypo) manic episodes (Grande, Berk, Birmaher, & Vieta, 2015; Kupka, Knoppert, & Nolen, 2008). In general, a distinction is made between bipolar I (BDI) and bipolar II (BDII) disorder (American Psychiatric Association, 2013). In BDII, a person experiences hypomanic and depressive episodes but never a full manic episode. Prevalence estimates reveal a lifetime prevalence of 0.6% for BDI and 0.4% for BDII (Merikangas et al., 2011). The economic burden of BD was estimated at 151 billion dollars per year in the United States (Dilsaver, 2011) and the illness is associated with decreased quality of life (Dean, Gerner, & Gerner, 2004), negative social consequences (Calabrese et al., 2003), work‐related problems (Fajutrao, Locklear, Priaulx, & Heyes, 2009; Laxman, Lovibond, & Hassan, 2008), and high caregiver burden (Miller, Dell'Osso, & Ketter, 2014; Reinares et al., 2006).

In mental health care, the importance of personal recovery is becoming increasingly emphasized in the treatment of patients with mood disorders (Fava, Ruini, & Belaise, 2007; Jones, Mulligan, Higginson, Dunn, & Morrison, 2013; Slade, 2010). Personal recovery can be defined as the ability to live a meaningful, hopeful, and contributing life, even in the presence of mental illness (Leamy, Bird, Le Boutillier, Williams, & Slade, 2011). Closely related to this conception is the notion of mental health (Keyes, 2002; Keyes, 2005), defined as the absence of psychological symptoms, but also as the presence of well‐being. Well‐being comprises an emotional component (feeling well, e.g., the presence of positive emotions) and a social (e.g., contribution to society) and psychological dimension (living well, e.g., personal growth). Research indicates that well‐being protects against the recurrence of psychopathology (Keyes, Dhingra, & Simoes, 2010; Lamers, Westerhof, Glas, & Bohlmeijer, 2015; Schotanus‐Dijkstra et al., 2016; Trompetter, de Kleine, & Bohlmeijer, 2017). Furthermore, social role participation is increasingly seen as an important factor of recovery and refers to the ability to fulfill social roles (Jaeger & Hoff, 2012; Whitley & Drake, 2010). Social role participation has been shown to be important for building and maintaining self‐esteem and autonomy (Gordeev et al., 2010) and contributes to long‐term mental health (Oude Voshaar et al., 2016).

Research emphasizes the relevance of cognitive emotion regulation processes for the onset and recurrence of mood disorders (Nolen‐Hoeksema, 1991). The impact of cognitive response to negative affect has been extensively studied (Aldao, Nolen‐Hoeksema, & Schweizer, 2010; Nolen‐Hoeksema, Wisco, & Lyubomirsky, 2008). However, less research has focused on the role of cognitive responses to positive affect, even though these processes might be equally important to understand emotion regulation processes in mood disorders (Carl, Soskin, Kerns, & Barlow, 2013; Wood, Heimpel, & Michela, 2003).

Two types of cognitive responses to positive affect appear especially relevant in the context of BD. Dampening refers to the suppression of positive affect to reduce the intensity of a positive mood (Quoidbach, Berry, Hansenne, & Mikolajczak, 2010). Positive rumination can be described as the tendency to respond to positive affective states with recurrent thoughts about positive experiences (Feldman, Joormann, & Johnson, 2008). Paradoxically, dampening is associated with risk for mania (Johnson, McKenzie, & McMurrich, 2008), and is heightened among people with BD (Edge et al., 2013; Johnson, Tharp, Peckham, & McMaster, 2016). Furthermore, dampening has been shown to predict depressive, and interestingly also manic symptoms over the course of 6 months in remitted persons with BD (Gilbert, Nolen‐Hoeksema, & Gruber, 2013). Dampening positive emotions may thus be a particular maladaptive strategy (Gilbert et al., 2013). In addition, dampening has been shown to have a negative impact on life satisfaction (Quoidbach et al., 2010) and correlates positively with depressive symptoms and self‐esteem (Feldman, Joormann, & Johnson, 2008; Raes, Daems, Feldman, Johnson, & Van Gucht, 2010). Positive rumination strategies have been shown to be related with increases in different aspects of well‐being, such as positive affect and life satisfaction (Quoidbach et al., 2010), lower levels of depressive symptoms (Feldman et al., 2008; Raes et al., 2010), but also with higher mania lifetime frequency (Gruber, Eidelman, Johnson, Smith, & Harvey, 2011) and risk for manic or hypomanic episodes (Johnson & Jones, 2009). Emotion‐focused positive rumination has been shown to be positively related to lifetime diagnoses of mania or hypomania and self‐focused positive rumination has been related to current manic symptom severity (Johnson et al., 2008).

To assess cognitive responses to positive affect, the Responses to Positive Affect (RPA) questionnaire has been developed (Feldman et al., 2008). An initial psychometric evaluation of the questionnaire in a student sample yielded three underlying factors: (a) dampening; (b) emotion‐focused positive rumination; and (c) self‐focused positive rumination (Feldman et al., 2008). Emotion‐focused positive rumination refers to rumination on positive moods and somatic experiences with the aim to intensify current positive mood states, while self‐focused rumination is signified by ruminating about positive qualities or personally relevant goals (Dempsey, Gooding, & Jones, 2011; Feldman et al., 2008; Olofsson, Boersma, Engh, & Wurm, 2014). The factor structure of the RPA has been replicated in a Dutch community sample (Raes et al., 2010) as well as Swedish (Olofsson et al., 2014) and Chinese (Yang & Guo, 2014) student samples. The RPA revealed convergent validity in relation to measures of emotion regulation and self‐esteem and incremental validity to depressive and manic symptoms.

Although responses to positive affect may be relevant for people with BD, there are some important gaps in current knowledge. The RPA has not been validated in a clinical sample of persons with BD. Also, the relations between responses to positive affect and constructs relating to personal recovery and well‐being have not been explored yet. The aim of the present study was to: (a) investigate the factor structure and internal consistency of the RPA in a clinical group of persons with BD; (b) study the construct validity of the RPA by investigating the relation with personal recovery, well‐being, social role participation, and symptomatology; and (c) determine the incremental validity of the RPA in explaining variance in personal recovery above and beyond measures of well‐being and social role participation.

Concerning the factor structure of the RPA, we hypothesized that the 3‐factor structure of the RPA found in earlier studies (Feldman et al., 2008; Nelis et al., 2016; Raes et al., 2010) will be replicated in the current clinical sample. With respect to construct validity, prior studies have shown negative associations between dampening and well‐being related outcomes, such as life satisfaction and self‐esteem (Quoidbach et al., 2010; Raes et al., 2010) and a positive relationship between positive rumination strategies and well‐being outcomes (Quoidbach et al., 2010). Hence, it was hypothesized that dampening would correlate weakly to moderately with well‐being. Although the relationship between positive emotion regulation and personal recovery has not been explored yet, well‐being and personal recovery share substantial conceptual overlap (Slade, 2010). Thus, we expected similar correlations with personal recovery. Since dampening might hamper people with BD to have a meaningful and contributing life, we expected a weak to moderate negative correlation between dampening and social role participation. Furthermore, we anticipated dampening and depressive symptoms to be moderately positively correlated (Feldman et al., 2008; Raes et al., 2010; Raes, Pommier, Neff, & Van Gucht, 2011) and expected a low to moderate correlation between dampening and anxiety symptoms, since dampening was moderately related with anxiety (Olofsson et al., 2014) and ruminative thinking and brooding (Feldman et al., 2008; Raes et al., 2010). Furthermore, we expected that both self‐focused and emotion‐focused positive rumination show small to moderate correlations with constructs of manic symptoms (Gruber et al., 2011; Johnson & Jones, 2009; Johnson et al., 2008). Finally, we assumed that positive rumination would reveal a positive small to moderate correlation with the constructs of personal recovery, well‐being, and social role participation (Quoidbach et al., 2010).

## 2 METHOD

### 2.1 Procedure

The study was approved by the Ethics Committee of the Faculty of Behavioral, Management, and Social Sciences of the University of Twente. Data were collected between April and July 2018. The survey was conducted via the online survey tool LimeSurvey (https://www.limesurvey.org/). Participants were gathered through convenience sampling via the Dutch patient association for BD, where the study was advertised through a notice in the newsletter of the patient association. Enrollment of participants was based on self‐selection. Diagnoses of the participants were self‐reported only and not confirmed by a clinical interview. At the start of the survey, participants were informed about the scope of the study and that participation was voluntary, could be stopped at any moment, and that data were processed anonymously and confidentially. Ten shopping vouchers of 50 euro were raffled among all participants.

### 2.2 Measures

Participants were asked to specify their gender, age, marital and employment status, ethnicity, and educational background and also their type of diagnosis (BDI or BDII). Moreover, they were asked to state whether they were in psychological or psychiatric treatment and if they were taking medications in the context of their BD. Finally, participants were asked whether there were any recent adaptations in their medication and if they experienced a relapse into a mood episode in the past 6 months. The following questionnaires were used to assess relevant constructs:

#### 2.2.1 Responses to positive affect

The RPA questionnaire (Feldman et al., 2008; Raes et al., 2010) consists of 17 items and measures cognitive responses to positive affective states. Respondents rate the items on a 4‐point Likert scale, ranging from 1 (almost never) to 4 (almost always). The RPA consists of three subscales: (a) emotion‐focused positive rumination (five items); (b) dampening (eight items); and (c) self‐focused positive rumination (four items). Cronbach's alpha (α) in a prior psychometric evaluation was 0.80 for the subscales self‐focused positive rumination and dampening, and 0.72 for the emotion‐focused positive rumination (Raes et al., 2010).

#### 2.2.2 Personal recovery

The 15‐item version of the Questionnaire about the Process of Recovery (QPR; Law, Neil, Dunn, & Morrison, 2014; Neil et al., 2009) was used to assess personal recovery. Items of the QPR are scored on a 5‐point Likert scale, ranging from 0 to (disagree strongly) to 4 (agree strongly) and higher total scores indicate better personal recovery. For the purpose of this study, the QPR was translated by the first and second author of this article into Dutch through forward and backward translation. The English 15‐item version of the QPR showed high internal consistency in a sample of psychotic individuals (α = 0.89; Williams et al., 2015) and in a sample of people with schizophrenia spectrum disorder (α = 0.93; Law et al., 2014). In the present study, Cronbach's α was 0.93.

#### 2.2.3 Well‐being

The Mental Health Continuum—Short Form (MHC‐SF; Lamers, Westerhof, Bohlmeijer, ten Klooster, & Keyes, 2011) is a 14‐item self‐report questionnaire assessing well‐being on three dimensions: (a) emotional well‐being (three items); (b) psychological well‐being (six items); and (c) social well‐being (five items). On a 6‐point Likert scale, respondents rate the frequency of feelings in the past month. For this study, the Dutch version of the MHC‐SF was used, which revealed high internal consistency for the subscales emotional (α = 0.83) and psychological well‐being (α = 0.83) and adequate reliability for social well‐being (α = 0.74; Lamers et al., 2011). Cronbach's α in the current study was 0.89, 0.87, and 0.67 for emotional, psychological, and social well‐being, respectively.

#### 2.2.4 Social role participation

Social role participation was assessed using the short version of the Social Role Participation Questionnaire (S‐SRPQ; Oude Voshaar et al., 2016). This 12‐item questionnaire measures the influence of mental health on six social roles along two dimensions: (a) satisfaction with the role; and (b) experienced psychological difficulty. Items are scored on a 5‐point Likert scale, reaching from 0 (not satisfied at all, respectively, no difficulties at all) to 4 (very much satisfied, respectively, not possible). Higher scores indicate more satisfaction and more experienced difficulties with a social role. A recent psychometric evaluation by Oude Voshaar et al. (2016) revealed high internal consistency for both subscales (α = 0.86). Cronbach's α in the current study was 0.75 and 0.82 for the subscales satisfaction with the role and experienced psychological difficulty, respectively.

#### 2.2.5 Depression and anxiety symptoms

The 14‐item Hospital Anxiety and Depression Scale (HADS; Zigmond & Snaith, 1983) assesses the presence of psychopathology in two domains: anxiety (seven items) and depressive symptoms (seven items). Respondents rate the frequency of symptoms over the last week from 0 (not at all) to 3 (very often)*,* with higher scores indicating more psychopathology. Scores of eight or higher on one of the subscales are seen as cut‐off score for caseness (Bjelland, Dahl, Haug, & Neckelmann, 2002). A psychometric evaluation by Spinhoven et al. (1997) found acceptable internal consistency for the depression subscale (α = 0.79) and good reliability for the anxiety subscale (α = 0.88). Cronbach's α in the current study was 0.73 for the depression and 0.85 for the anxiety subscale.

#### 2.2.6 Manic symptoms

The Altman Self‐Rating Mania Scale (ASRM; Altman, Hedeker, Peterson, & Davis, 1997) is a 5‐item self‐rating scale measuring symptoms of mania in the past week. Items include symptoms of mania (e.g., inflated self‐confidence). Each item provides five response options with increasingly severe descriptions. Total scores are calculated by summing up the scores on each item and higher scores indicate more manic symptoms. Scores of six or higher are an indication of the presence of meaningful manic symptoms (Altman et al., 1997). The ASRM has been shown to have high test‐retest reliability (Altman et al., 1997), to be sensitive to changes in a clinical group (Altman, Hedeker, Peterson, & Davis, 2001) and to predict related outcomes in student samples (Meyer, Beevers, & Johnson, 2004). Cronbach's α was 0.73 in the current study.

## 3 STATISTICAL ANALYSES

Statistical analyses were performed using Mplus version 7.11 (Muthén & Muthén, 2010), RStudio (R Core Team, 2018) and the statistical package for social sciences version 25 (SPSS). First, confirmatory factor analyses (CFA) were conducted with Mplus to investigate whether the dimensionality of the RPA found in earlier studies in nonclinical samples could be confirmed in the current BD sample. Therefore, we fitted the original 3‐factor model, in which five items load on the emotion‐focused positive rumination factor, four items on the self‐focused positive rumination factor, and eight items on the dampening factor. Afterward, we tested a 2‐factor model, in which the nine items of the two positive rumination subscales loaded on a single latent positive rumination factor and eight items on the dampening factor. The fit of the models was based on the restrictive assumption that error terms of items were uncorrelated. Since correlated error terms are indicative of model misspecification, but are not unusual in psychological assessment instruments (Byrne, Baron, Larsson, & Melin, 1995), we decided to only allow error correlations if this made substantive sense and if none of the restrictive models achieved acceptable fit (Jöreskog, 1993). Considering the ordinal nature of the data and the small sample size, we followed the recommendations by Flora and Curran (2004) and Moshagen and Musch (2014) and used a robust diagonally weighted least square mean and variance adjusted (WLSMV) estimation method. In the literature, factor loadings higher than 0.30 or 0.40 are usually seen as satisfactory (Floyd & Widaman, 1995; Hair, Black, Babin, Anderson, & Tatham, 2009) and we used >0.35 as a compromise. Model fit for both models was assessed using chi‐square (*χ* ^2^) statistics, in which a smaller value indicates better model fit and the ratio between *χ* ^2^ and degrees of freedom should be <3 for an acceptable fit (Kline, 2015). Furthermore, the comparative fit index (CFI), Tucker‐Lewis Index (TLI), weighted root‐mean‐square residual (WRMR), and root‐mean‐square error approximation (RMSEA) were calculated to determine the model fit (Hu & Bentler, 1998). Values ≥0.90 were seen as acceptable and values ≥0.95 as good model fit for the CFI and TLI, whereas RMSEA values ≤0.80 and ≤0.50 were considered as acceptable and good model fit, respectively (Browne & Cudeck, 1992; Hu & Bentler, 1999). For the WRMR, estimates around one were seen as good model fit (DiStefano, Liu, Jiang, & Shi, 2018; Yu, 2002). The difference in fit between the 3‐factor and 2‐factor models was statistically tested using the Mplus DIFFTEST procedure, which computes differences in *χ* ^2^ values of nested models.

Internal consistency was evaluated by calculating Cronbach's α and categorical McDonald's omega (ω) for the subscales of the 2‐factor and 3‐factor‐models (Dunn, Baguley, & Brunsden, 2014). McDonald's ω deals with the limitation of Cronbach's α, which assumes tau‐equivalence and thus shows deficiencies for congeneric models (Kelley & Pornprasertmanit, 2016). Using the MBESS package (Kelley, 2018), α and ω coefficients with 95% bias‐corrected and accelerated bootstrap confidence intervals based on 10,000 bootstrap samples were calculated. The α and ω estimates >0.70 and >0.80 were seen as acceptable and good internal consistency (Cicchetti, 1994).

For examining construct validity, bivariate Pearson's correlation coefficients were calculated between the three subscales of the RPA and criterion measures. Correlation coefficients between 0.1 and 0.3 were interpreted as weak, coefficients larger than 0.3 and smaller than 0.5 as moderate and larger or equal to 0.5 as strong correlations (Cohen, 1988). To determine incremental validity of the RPA in explaining variance in personal recovery, multiple hierarchical regression analyses were performed with total scores of the QPR as the dependent variable. In the first step, scores of the MHC‐SF, S‐SRPQ, HADS, and ASRM were entered, respectively. Scores of the subscales of the RPA were entered in Step 2. Significant changes in explained variance after the second step (*p* < .05) were seen as indicative for incremental validity.

## 4 RESULTS

### 4.1 Description of the sample

Mean age of the 107 participants was 52 years (SD = 11.26, range 23–77). Of the sample, 55.1% (*n* = 59) experienced a relapse into a depressive or manic episode in the past 6 months and 50.5% had adaptations to their medications in the past 6 months (*n* = 54). Eight participants were administered to a psychiatric hospital due to symptoms related to BD. Of the participants who completed the HADS, 61 (62.2%) scored above the clinical threshold for anxious symptomatology and 68 (69.4%) for depression. For manic symptomatology, 20 (20.4%) participants scored above the cut‐off for manic symptomatology. Sample characteristics are summarized in Table 1.

**Table 1:** Sample characteristics (*N* = 107)

		N	%
Age	Mean = 52 (range 23–77)		
Gender	Female	82	76.6
	Male	25	23.4
Marital status	Married	56	52.3
	Never married	28	26.6
	Divorced	22	20.6
	Widowed	1	0.9
Employment status	Unable to work	39	36.4
	Paid work	26	24.3
	Unpaid/voluntary work	14	13.1
	Retired	10	9.3
	Housewife/houseman	5	3.7
	Self‐employed	4	4.7
	Student	3	2.8
	Other	6	5.6
Education	Low	14	13.2
	Moderate	35	33.1
	High	57	53.7
Diagnosis	BDI	42	39.3
	BDII	51	47.7
	Unknown	14	13.0
Relapse into mood episode (past 6 months)	Yes	59	55.1
	No	48	44.9
Currently in psychological of psychiatric treatment	Yes	89	83.2
	No	18	16.8
Currently taking medication	Yes	92	95.3
	No	5	4.7

Abbreviations: BDI, bipolar I; BDII, bipolar II.

### 4.2 Factor structure and internal consistency

An initial CFA with the original 3‐factor and 2‐factor models revealed a very low factor loading of 0.13 in both models for item number 6 (“Think this is too good to be true”). We decided to drop this item for further analyses, a strategy which is in line with earlier studies (Kim & Kwon, 2014; Nelis et al., 2016). Afterward, we again fitted a 2‐factor and 3‐factor model, in which only seven items load on the latent dampening factor. Fit indices for the 2‐factor and 3‐factor models without item 6 are presented in Table 2.

**Table 2:** Goodness of fit indices for two models tested in CFA

Model	*χ* ^2^	*df*	CFI	TLI	WRMR	RMSEA (90% CI)
2‐factor model	194.70	103	0.908	0.893	1.098	0.091 (0.071–0.111)
3‐factor model	181.55	101	0.919	0.904	1.032	0.086 (0.066–0.106)

*Note: N*  = 107.

Abbreviations: CFI, comparative fit index; CI, confidence interval; df,  degrees of freedom; RMSEA,  root‐mean‐square error of approximation; TLI, tucker‐lewis index; WRMR, weighted root‐mean‐square residual; *χ* ^2^,  chi‐square statistics.

Overall model fit indices were slightly better for the 3‐factor model. This was confirmed by the *χ* ^2^ test for difference testing, which revealed that the two models significantly differed in their fit (*Δχ* ^2^ = 11.32; *Δdf *=* *2; *p < *.01). CFI estimates were acceptable for both tested models, but the TLI was acceptable only for the 3‐factor model. The ratio between *χ* ^2^ and degrees of freedom was <2 for both models, indicating a good fit. For the 2‐factor and 3‐factor models, WRMR estimates (1.098 and 1.032, respectively) and RSMEA values (0.091 and 0.086, respectively) fell short of the criterion for adequate fit. However, it should be noted that the WRMR is an experimental test statistic and cutoffs for fit are still debated. Modification indices revealed an improvement of the 3‐factor model, if the error correlation between item 10 (“Remind yourself that these feelings won’t last”) and 15 (“I am lucky for now, but this will end soon”) was allowed (*r* = 0.647). In this model, CFI and TLI values increased to 0.924 and 0.938, respectively, RMSEA estimates improved to 0.076 and the *χ* ^2^ value decreased (*χ* ^2^ = 161.80; *df *=* *100; *p < *.001). Besides the WRMR, all fit indices revealed an adequate fit in this model and the *χ* ^2^ test for difference indicated a significantly better fit of the 3‐factor model with error correlation compared with the original 3‐factor model without error correlation (*Δχ* ^2^ = 19.75; *Δdf *=* *1; *p < *.001). However, since the 3‐factor model without error correlation was already showing acceptable fit, we decided to adhere to this model and not allow error correlations for further analyses.

Standardized factor loadings for the 3‐factor model (without error correlation) and 2‐factor model and corresponding Cronbach's α and categorical McDonald's ω coefficients are shown in Table 3. All standardized factor loadings were above 0.35 and McDonalds's ω coefficients revealed good and adequate internal consistency for dampening (*ω* = 0.86), emotion‐focused (*ω* = 0.78), and self‐focused positive rumination (*ω *=* *.77).

**Table 3:** Standardized factor loadings for the two tested models and corresponding Cronbach's alpha and categorical McDonald's omega for subscales of the RPA

RPA Item	3‐factor model			2‐factor model	
	D	EF	SF	D	PR
Dampening					
Think about things that could go wrong (rpa9)	0.57	–	–	0.58	–
Remind yourself that these feelings won’t last (rpa10)	0.77	–	–	0.78	–
Think “People will think I am bragging.” (rpa11)	0.54	–	–	0.53	–
Think about how hard it is to concentrate (rpa12)	0.63	–	–	0.62	–
Think “I don’t deserve this.” (rpa14)	0.65	–	–	0.64	–
Think “I am lucky for now, but this will end soon.” (rpa15)	0.84	–	–	0.84	–
Think about the things that have not gone well for you (rpa17)	0.66	–	–	0.66	–
Emotion‐focus					
Notice how you feel full of energy (rpa1)	–	0.72	–	–	0.69
Focus on enjoying this moment (rpa2)	–	0.73	–	–	0.68
Think about how happy you feel (rpa7)	–	0.48	–	–	0.45
Think about how strong you feel (rpa8)	–	0.78	–	–	0.75
Think about how proud you are of yourself (rpa16)	–	0.71	–	–	0.68
Self‐focus					
Think “I am getting everything done.” (rpa3)	–	–	0.84	–	0.82
Think how you feel ready to do anything (rpa4)	–	–	0.82	–	0.77
Think “I am the best I could be.” (rpa5)	–	–	0.64	–	0.61
Think “I am achieving everything I could want.” (rpa13)	–	–	0.80	–	0.77
Cronbach's alpha (95% CI)	0.80 (0.73−0.86)	0.77 (0.69−0.83)	0.75 (0.65−0.82)	0.80 (0.73−0.86)	0.85 (0.80−0.89)
McDonald's omega (95% CI)	0.86 (0.74−0.91)	0.78 (0.66−0.85)	0.77 (0.65−0.83)	0.86 (0.74−0.91)	0.89 (0.80−0.93)

Abbreviations: CI, confidence interval; D, dampening; EF, emotion‐focused positive rumination; PR, positive rumination; RPA, responses to positive affect; SF, self‐focused positive rumination.

### 4.3 Construct validity

Table 4 presents the mean values and bivariate intercorrelations between the subscales of the RPA and criterion measures. Dampening was weakly negatively associated with emotional, psychological well‐being, and overall well‐being. Higher levels of dampening were found to be moderately associated with the less personal recovery and with more anxiety symptoms. Scores of emotion‐focused positive rumination were moderately positively correlated with all facets of well‐being and also with personal recovery. Weak negative correlations were found between emotion‐focused positive rumination and experienced difficulty with social roles and anxiety symptoms and a moderate negative association with depressive symptoms. No significant relation was found between self‐focused positive rumination and the subscales emotional (*p* = .06), social (*p* = .11), and psychological well‐being (*p* = .06). Surprisingly, self‐focused positive rumination was moderately associated with both higher levels of personal recovery and symptoms of mania.

**Table 4:** Descriptive statistics and bivariate correlations between the RPA and criterion measures

Measure	M (SD)	Dampening	Emotion‐focus	Self‐focus
RPA (*n* = 107)				
Dampening	13.35 (3.98)	–	–	–
Emotion‐focus	12.30 (2.90)	−0.26**	–	–
Self‐focus	8.21 (2.63)	−0.15	0.62**	–
MHC‐SF (*n* = 102)				
Emotional well‐being	7.40 (3.96)	−0.22*	0.42**	0.19
Social well‐being	8.36 (4.94)	−0.18	0.34**	0.16
Psychological well‐being	13.58 (7.19)	−0.21*	0.37**	0.19
Total score	29.34 (14.48)	−0.20*	0.41**	0.20*
QPR (*n* = 102)				
Total score	37.66 (11.14)	−0.33**	0.42**	0.31**
S‐SRPQ (*n* = 98)				
Satisfaction with role	14.62 (5.41)	−0.17	0.21	0.11
Experienced difficulty	16.77 (5.41)	0.20*	−0.22*	−0.08
HADS (*n* = 98)				
Anxiety symptoms	8.71 (4.64)	0.47**	−0.24*	−0.04
Depression symptoms	9.61 (3.99)	0.20*	−0.39**	−0.19
ASRM (*n = *98)				
Total score	2.99 (3.23)	0.14	0.19	0.34**

*Note*: Variations in *n* due to missing data.

Abbreviations: ASRM,  altman self‐rating mania scale; HADS,  hospital anxiety and depression scale; MHC‐SF,  mental health continuum—short form; QPR , questionnaire about the process of recovery; RPA, responses to positive affect questionnaire (without item 6): S‐SRPQ, short version of the social role participation questionnaire.

* *p* < .05

** *p* < .01.

### 4.4 Incremental validity

To determine incremental validity of the RPA, we conducted several multiple hierarchical regressions analyses with scores of the QPR as the criterion variable. The RPA explained 4% additional variance in personal recovery above and beyond measures of well‐being (*p* < .01). Dampening and self‐focused positive rumination significantly explained variance in personal recovery independently of the MHC‐SF subscales. Furthermore, the RPA explained 14% additional variance in personal recovery above and beyond social role participation (*p* < .001). Dampening significantly explained variance above and beyond personal recovery independent of the scores of social role participation (*p* < .05). Findings regarding incremental validity are summarized in Table 5 and Table 6.

**Table 5:** Summary of hierarchical regression analysis for MHC‐SF and RPA subscales and personal recovery (QPR)

Variable	*B*	*SE*	*β*	*t*	*ΔR* ^2^
Step 1					
Constant	19.17	1.43		13.44***	0.69***
Emotional well‐being (MHC‐SF)	1.08	0.25	0.39	4.31***	
Psychological well‐being (MHC ‐SF)	0.75	0.17	0.49	4.52***	
Social well‐being (MHC‐SF)	0.03	0.19	0.01	0.15	
Step 2					
Constant	21.80	3.77		5.47***	0.04**
Emotional well‐being (MHC‐SF)	1.02	0.25	0.36	4.16***	
Psychological well‐being (MHC ‐SF)	0.72	0.16	0.46	4.53***	
Social well‐being (MHC‐SF)	0.02	0.18	0.01	0.10	
Dampening (RPA)	−0.38	0.15	−0.14	−2.48*	
Emotion‐focus (RPA)	−0.18	0.29	−0.05	−0.62	
Self‐focus (RPA)	0.69	0.29	0.17	2.42*	

*Note*: MHC‐SF, mental health continuum—short form; QPR, questionnaire about the process of recovery; RPA, responses to positive affect questionnaire.

* *p* < .05

** *p* < .01

*** *p* < .001.

**Table 6:** Summary of hierarchical regression analysis for S‐SRPQ and RPA subscales and personal recovery (QPR)

Variable	*B*	*SE*	*β*	*t*	*ΔR* ^2^
Step 1					
Constant	26.28	6.84		3.84***	0.41***
Satisfaction with a role (S‐SRPQ)	1.12	0.25	0.52	4.74***	
Experienced difficulty (S‐SRPQ)	−0.34	0.22	−0.16	−1.50	
Step 2					
Constant	19.38	7.62		2.73**	0.14***
Satisfaction with a role (S‐SRPQ)	1.07	0.22	0.47	4.87***	
Experienced difficulty (S‐SRPQ)	−0.22	0.20	−0.11	−1.08	
Dampening (RPA)	−0.52	0.20	−0.19	−2.62*	
Emotion‐focus (RPA)	0.65	0.35	0.17	1.90	
Self‐focus (RPA)	0.65	0.37	0.16	1.78	

Abbreviations: QPR, questionnaire about the process of recovery; RPA, responses to positive affect questionnaire; S‐SRPQ, social role participation questionnaire.

* *p* < .05

** *p* < .01

*** *p* < .001.

In addition, we investigated whether the RPA explained variance in personal recovery above and beyond measures of symptomatology. The RPA explained 9.6% additional variance above and beyond depressive symptoms (F change [196] = 7.21; *p* < .001; adjusted *R* ^2^ Step 2 = 0.58). More specifically, self‐focused positive rumination (*p* < .05) and dampening (*p* < .001) independently explained personal recovery above and beyond depressive symptomatology. The RPA explained 17.5% additional variance in personal recovery outcomes above and beyond anxiety symptoms (F change [196] = 8.85; *p* < .001; adjusted *R* ^2^ Step 2 = 0.37). Only self‐focused positive rumination independently explained variance in personal recovery above anxious symptomatology (*p* < 0.01). Finally, the RPA explained 27.4% additional variance above and beyond ASRM scores (F change [196] = 12.46; *p* < .001; adjusted *R* ^2^ Step 2 = 0.29). In this model, dampening (*p* < .001) and emotion‐focused positive rumination (*p* < .05) independently explained variance in personal recovery above and beyond manic symptomatology.

## 5 DISCUSSION

The present study set out to evaluate the psychometric properties of the RPA in a clinical sample of persons with BD. The RPA was designed as a measure of positive emotion regulation (Feldman et al., 2008). Growing evidence of responses to positive affect in mental disorders emphasizes the importance of these processes in mood disorders (Carl et al., 2013; Edge et al., 2013; Gruber et al., 2011; Johnson et al., 2008). Several prior studies evaluated the psychometric properties of the RPA (Feldman et al., 2008; Kim & Kwon, 2014; Olofsson et al., 2014; Raes et al., 2010; Yang & Guo, 2014), but used community or student samples.

Results of the CFA showed that the 3‐factor model had a significantly better fit to the data than the 2‐factor model, coinciding with prior studies (Feldman et al., 2008; Raes et al., 2010; Yang & Guo, 2014). Most fit indices showed an adequate fit, but the RMSEA fell short of the criterion for acceptable fit. However, it should be noted that fit indices are differentially sensitive to the type of model and several misspecifications. For this reason, Hu and Bentler (1998) recommend to report several different fit indices to assess the overall quality of a model. The relevance of distinguishing between emotion‐focused and self‐focused positive rumination was also apparent from their differential associations with criterion measures. In the course of the CFA, we decided to remove item number 6 of the RPA (“This is too good to be true”) from the further analyses. One possible explanation for the low factor loadings of this item might be the position in the questionnaire since the five items before and the two items after the item belong to positive rumination. Alternatively, the formulation of the item could be considered in a nondampening manner (Nelis et al., 2016).

Results of the correlational analyses showed that more dampening was moderately associated with the reduced personal recovery and weakly with lower well‐being, coinciding with our hypotheses (Quoidbach et al., 2010). This finding can be interpreted as dampening being an obstacle for people with BD to reach personal recovery. Mansell (2016) argues that the fear of feeling too good and becoming manic might be one of the greatest problems in BD. This fear might lead to dampening and inhibit people from leading a meaningful life. This hypothesis is supported by a moderate correlation between dampening and anxious symptoms in the present study. Moreover, earlier studies suggest that persons with BD tend to avoid rewarding activities to prevent mania (Edge et al., 2013) and that dampening is associated with poorer well‐being (Quoidbach et al., 2010) and quality of life (Edge et al., 2013). Interestingly, our results revealed only a weak relationship between dampening and depressive symptoms. It would be interesting for future research to specifically explore the role of dampening in relation to well‐being and symptomatology in this group.

Emotion‐focused positive rumination was moderately correlated with measures of well‐being and personal recovery, which is in line with earlier research (Gilbert et al., 2013; Quoidbach et al., 2010). We found a nonsignificant relationship between emotion‐focused positive rumination and manic symptoms, but a significant relationship with more anxious and depressive symptoms. Interestingly, for self‐focused positive rumination, this relationship was inverted, as it showed a significant relationship with manic symptoms, but not with depressive or anxiety symptoms. One possible explanation for this finding might be that self‐focused positive rumination is associated with manic symptoms because it focusses on personal qualities and specific actions and, while emotion‐focused positive rumination specifically focusses on affective processes. Also, the low proportion of participants meeting the threshold for manic symptoms might be a possible explanation for some of the null results around manic symptoms.

Scores of the RPA showed incremental validity by explaining variance in personal recovery above and beyond well‐being. This is a remarkable finding with respect to the utility of the RPA since the concept of well‐being shares several commonalities with personal recovery (Slade, 2010). Specifically, dampening and self‐focused positive rumination explained additional variance in personal recovery. This supports the assumption that tackling dampening might be an effective way to improve personal recovery. We also found that dampening explained variance in personal recovery above and beyond social role participation, again underscoring the importance of dampening in relation to personal recovery.

### 5.1 Implications

Some relevant implications for both research and clinical practice arise from these findings. First, when using the RPA for assessment purposes, caution should be paid to item number 6. We advise that this item is not used in calculating the dampening subscale score. Future research could examine if adjusting the position of the item in the questionnaire results in a better fit with its assumed factor. Since the dampening subscale also revealed good internal consistency without this item, it could also be considered to remove the item altogether. Second, our findings suggest that positive emotion regulation is an important factor in BD. Health care professionals working with patients with BD should try to tackle the tendency to dampen positive emotions and at the same time try to foster positive rumination, especially when aiming to achieve personal recovery and improved well‐being.

### 5.2 Limitations and future research

Our study has several limitations, giving opportunities for future research. First, the sample size was relatively small. For example, Kline (2015) recommends a sample of at least 200 participants for sound structural equation modeling procedures. Although we tried to compensate for the relatively small sample by using WLSMV estimation, the modest sample might have distorted the fit indices of the factor models. Future research could investigate if the superiority of the 3‐factor model can be confirmed and whether the factor structure is invariant across subgroups of patients. Second, no conclusions about causality can be drawn from the study. For example, it cannot be concluded that dampening actually leads to less personal recovery. A reverse causal direction might also be plausible. Future research should explore the relationship between these concepts in a longitudinal design. Third, the diagnosis of BD was based on self‐report, which leaves the possibility that not all participants had a clinically confirmed diagnosis of BD. However, since participants were recruited via the patient association for BD, most people indicated a specific type of BD and 95% of the sample stated that they were taking the medication in the context of their BD, it can be assumed that the vast majority of the sample actually had BD.

## 6 CONCLUSIONS

Our findings suggest that the RPA is an internally consistent and valid tool to assess positive emotion regulation processes in persons with BD. Furthermore, dampening and positive rumination seem to play an important role in BD patients and specifically in relation to personal recovery and well‐being. This relationship deserves closer examination in the context of longitudinal studies.
